# Supplementary material for: HIVprotI: an integrated web based platform for prediction and design of HIV proteins inhibitors
Source: J Cheminform. 2018 Mar 9;10:12. doi: 10.1186/s13321-018-0266-y (PMC5845081; doi:10.1186/s13321-018-0266-y)
Supplement: Supplementary file 2 — Additional file 2. Source code of HIVProtI web server. [file 13321_2018_266_MOESM2_ESM.zip › HIVprotI_Source-code/hivprotI/weka-3-6-6/doc/weka/core/converters/C45Loader.html]

C45Loader


---


|  |  |  |
| --- | --- | --- |
| **Overview** \| **Package** \| **Class** \| **Tree** \| **Deprecated** \| **Index** \| **Help** \| **Weka's home** | |  |
| **PREV CLASS**   **NEXT CLASS** | **FRAMES**    **NO FRAMES**     **All Classes** |  |
| SUMMARY: NESTED \| FIELD \| CONSTR \| METHOD | DETAIL: FIELD \| CONSTR \| METHOD |  |


---


## weka.core.converters Class C45Loader

```
java.lang.Object
  weka.core.converters.AbstractLoader
      weka.core.converters.AbstractFileLoader
          weka.core.converters.C45Loader
```

**All Implemented Interfaces:**: java.io.Serializable, BatchConverter, FileSourcedConverter, IncrementalConverter, Loader, EnvironmentHandler, RevisionHandler

---

``` public class C45Loader extends AbstractFileLoader implements BatchConverter, IncrementalConverter ```

Reads a file that is C45 format. Can take a filestem or filestem with .names or .data appended. Assumes that path/<filestem>.names and path/<filestem>.data exist and contain the names and data respectively.

**Version:**
:   $Revision: 1.16 $

**Author:**
:   Mark Hall (mhall@cs.waikato.ac.nz)

**See Also:**: `Loader`, Serialized Form

---

| **Field Summary** | |
| --- | --- |
| `static java.lang.String` | `FILE_EXTENSION`             the file extension |

**Fields inherited from class weka.core.converters.AbstractFileLoader**

`FILE_EXTENSION_COMPRESSED`

**Fields inherited from interface weka.core.converters.Loader**

`BATCH, INCREMENTAL, NONE`


**Constructor Summary**

`C45Loader()`


| **Method Summary** | |
| --- | --- |
| `Instances` | `getDataSet()`             Return the full data set. |
| `java.lang.String` | `getFileDescription()`             Returns a description of the file type. |
| `java.lang.String` | `getFileExtension()`             Get the file extension used for arff files |
| `java.lang.String[]` | `getFileExtensions()`             Gets all the file extensions used for this type of file |
| `Instance` | `getNextInstance(Instances structure)`             Read the data set incrementally---get the next instance in the data set or returns null if there are no more instances to get. |
| `java.lang.String` | `getRevision()`             Returns the revision string. |
| `Instances` | `getStructure()`             Determines and returns (if possible) the structure (internally the header) of the data set as an empty set of instances. |
| `java.lang.String` | `globalInfo()`             Returns a string describing this attribute evaluator |
| `static void` | `main(java.lang.String[] args)`             Main method for testing this class. |
| `void` | `reset()`             Resets the Loader ready to read a new data set or the same data set again. |
| `void` | `setSource(java.io.File file)`             Resets the Loader object and sets the source of the data set to be the supplied File object. |

**Methods inherited from class weka.core.converters.AbstractFileLoader**

`getUseRelativePath, retrieveFile, runFileLoader, setEnvironment, setFile, setUseRelativePath, useRelativePathTipText`

**Methods inherited from class weka.core.converters.AbstractLoader**

`setRetrieval, setSource`

**Methods inherited from class java.lang.Object**

`equals, getClass, hashCode, notify, notifyAll, toString, wait, wait, wait`

**Field Detail**

### FILE\_EXTENSION

```
public static java.lang.String FILE_EXTENSION
```

:   the file extension


**Constructor Detail**

### C45Loader

```
public C45Loader()
```


**Method Detail**

### globalInfo

```
public java.lang.String globalInfo()
```

:   Returns a string describing this attribute evaluator

    :   **Returns:**: a description of the evaluator suitable for displaying in the explorer/experimenter gui

---


### reset

```
public void reset()
           throws java.io.IOException
```

:   Resets the Loader ready to read a new data set or the
    same data set again.

    :   **Specified by:**: `reset` in interface `Loader` **Overrides:**: `reset` in class `AbstractFileLoader`
    :   **Throws:**: `java.io.IOException` - if something goes wrong

---


### getFileExtension

```
public java.lang.String getFileExtension()
```

:   Get the file extension used for arff files

    :   **Specified by:**: `getFileExtension` in interface `FileSourcedConverter`
    :   **Returns:**: the file extension

---


### getFileExtensions

```
public java.lang.String[] getFileExtensions()
```

:   Gets all the file extensions used for this type of file

    :   **Specified by:**: `getFileExtensions` in interface `FileSourcedConverter`
    :   **Returns:**: the file extensions

---


### getFileDescription

```
public java.lang.String getFileDescription()
```

:   Returns a description of the file type.

    :   **Specified by:**: `getFileDescription` in interface `FileSourcedConverter`
    :   **Returns:**: a short file description

---


### setSource

```
public void setSource(java.io.File file)
               throws java.io.IOException
```

:   Resets the Loader object and sets the source of the data set to be
    the supplied File object.

    :   **Specified by:**: `setSource` in interface `Loader` **Overrides:**: `setSource` in class `AbstractFileLoader`
    :   **Parameters:**: `file` - the source file. **Throws:**: `java.io.IOException` - if an error occurs

---


### getStructure

```
public Instances getStructure()
                       throws java.io.IOException
```

:   Determines and returns (if possible) the structure (internally the
    header) of the data set as an empty set of instances.

    :   **Specified by:**: `getStructure` in interface `Loader` **Specified by:**: `getStructure` in class `AbstractLoader`
    :   **Returns:**: the structure of the data set as an empty set of Instances **Throws:**: `java.io.IOException` - if an error occurs

---


### getDataSet

```
public Instances getDataSet()
                     throws java.io.IOException
```

:   Return the full data set. If the structure hasn't yet been determined
    by a call to getStructure then method should do so before processing
    the rest of the data set.

    :   **Specified by:**: `getDataSet` in interface `Loader` **Specified by:**: `getDataSet` in class `AbstractLoader`
    :   **Returns:**: the structure of the data set as an empty set of Instances **Throws:**: `java.io.IOException` - if there is no source or parsing fails

---


### getNextInstance

```
public Instance getNextInstance(Instances structure)
                         throws java.io.IOException
```

:   Read the data set incrementally---get the next instance in the data
    set or returns null if there are no
    more instances to get. If the structure hasn't yet been
    determined by a call to getStructure then method should do so before
    returning the next instance in the data set.
    If it is not possible to read the data set incrementally (ie. in cases
    where the data set structure cannot be fully established before all
    instances have been seen) then an exception should be thrown.

    :   **Specified by:**: `getNextInstance` in interface `Loader` **Specified by:**: `getNextInstance` in class `AbstractLoader`
    :   **Parameters:**: `structure` - the dataset header information, will get updated in case of string or relational attributes **Returns:**: the next instance in the data set as an Instance object or null if there are no more instances to be read **Throws:**: `java.io.IOException` - if there is an error during parsing

---


### getRevision

```
public java.lang.String getRevision()
```

:   Returns the revision string.

    :   **Specified by:**: `getRevision` in interface `RevisionHandler`
    :   **Returns:**: the revision

---


### main

```
public static void main(java.lang.String[] args)
```

:   Main method for testing this class.

    :   **Parameters:**: `args` - should contain <filestem>[.names | data]


---


|  |  |  |
| --- | --- | --- |
| **Overview** \| **Package** \| **Class** \| **Tree** \| **Deprecated** \| **Index** \| **Help** \| **Weka's home** | |  |
| **PREV CLASS**   **NEXT CLASS** | **FRAMES**    **NO FRAMES**     **All Classes** |  |
| SUMMARY: NESTED \| FIELD \| CONSTR \| METHOD | DETAIL: FIELD \| CONSTR \| METHOD |  |


---
